# Phenol-Formaldehyde Resin for Optical-Chemical Temperature Sensing

**DOI:** 10.3390/s18061756

**Published:** 2018-05-30

**Authors:** Steven Claucherty, Hirotaka Sakaue

**Affiliations:** Department of Aerospace and Mechanical Engineering, University of Notre Dame, Hessert Laboratory, Notre Dame, IN 46556, USA; sclauche@nd.edu

**Keywords:** phenol-formaldehyde resin, optical diagnostics, surface temperature measurement, temperature-sensitive paint, heat transfer

## Abstract

The application of phenol-formaldehyde (PF) resin as an optical temperature sensor is investigated. Recent developments in optical luminescent sensors allow for global measurements to be made over the surface of a test article, extending beyond conventional point measurements. Global temperature distributions are particularly helpful when validating computational models or when mapping temperature over complex geometries, and can be used to calculate surface heat flux values. Temperature-sensitive paint (TSP) is a novel chemical approach to obtaining these global temperature measurements, but there are still challenges to overcome to make it a reliable tool. A sensor with a wide range of temperature sensitivity is desired to provide the maximum amount of utility, especially for tests spanning large temperature gradients. Naturally luminescent materials such as PF resin provide an attractive alternative to chemical sensor coatings, and PF resin is studied for this reason. Static tests of different PF resin samples are conducted using two binder materials to strengthen the material: cloth and paper. The material shows temperature sensitivities up to −0.8%/K, demonstrating the usefulness of PF resin as a temperature sensor.

## 1. Introduction

In many scientific studies, understanding of heat transfer during a process or on model is crucial to understand the underlying thermodynamic processes. To fully understand the heat transfer, accurate temperature sensors must be used. Traditionally, these sensors have been limited to mechanical point sensors such as thermocouples. These provide wide temperature ranges, from cryogenic temperatures up to 2600 °C depending on the thermocouple type, and response times on the order of milliseconds with small diameter sensors; however, they are point sensors that limit the collected data to a series of local points. This study investigates the use of an alternative measurement technique using an optical-chemical sensor, which allows for similar temporal resolution to point sensors, but vastly improved spatial resolution. Advanced materials used in luminescent sensors have led to global sensors that can measure surface temperatures and pressures in a non-intrusive manner [1,2,3]. Temperature-sensitive paint (TSP) is an effective tool to measure the global temperature distribution over a body undergoing evaluation, most notably aerodynamic testing, and has seen use in high-speed testing up to 10 Mach [4,5,6]. The coating consists of a luminophore, also called an indicator dye, that responds to changes in temperature, and a binder material such as a polymer to adhere the coating to the model surface. The development of TSP and similar luminescent sensors are covered in literary reviews [2,7,8,9,10,11]. Current TSPs are applied to a test article via spraying or coating. This creates difficulties estimating the thermodynamic effects, such as local heat transfer, because of the added layer of material with its own thermal conductivity that must be considered. Traditional TSPs can also induce errors into these measurements due to imperfections or unevenness in the coating, resulting in additional surface roughness which can affect the flow conditions around the test model. In high speed testing, the durability of the sensor is another area of concern, which makes a sensor that does not require coating attractive. To make measurements in a cryogenic environment, a sensor is needed that functions well in temperatures approaching 100 K and below. Conversely, in very hot or high-speed flows, a sensor that can withstand temperatures exceeding 500 K is necessary. Commercial TSPs generally have temperature sensitivities between −0.9%/K and −3.68%/K, and research TSPs have ranges between −0.4%/K and −5.5%/K according to Sullivan and Liu; these paints tended to have small temperature ranges over which they provided high sensitivity [1]. Recent publications indicate research stage TSPs with larger effective temperature ranges, with sensitivities from −0.9%/K to −3.16%/K [12,13,14]. However, many of these coatings have narrow operating ranges, and it would be ideal if one sensor could be used over a wide range of temperatures for both types of tests; providing a wide range of temperature sensitivity with a sensor that does not need to be sprayed onto a model is the motivation for this PF resin-based temperature sensor.

Phenol-formaldehyde (PF) resin is a pure phenolic resin with an internal network of three-dimensional crosslinks; it is formed when phenol (C_6_H_5_OH) and formaldehyde (CH_2_O) undergo a condensation reaction, linking the phenol molecules with methylene bridges [15]. This gives the material the ability to resist deformation when it is heated, which is a clear advantage for a temperature sensor. Other advantages of PF resin are that it is naturally luminescent, inexpensive, easy to machine, nontoxic, and allows a model to have constant thermal properties. The density of PF resin is 1300 kg/m^3^, with a thermal conductivity and specific heat capacity of approximately 0.2 W/(m·K) and 0.92 kJ/(kg·K), respectively [16]. However, one drawback to using PF resin for model construction is its lower structural strength compared to stainless steel, which is typically used for aerodynamic models. For simple models or models in short duration testing, this would not necessarily present any issues; the sensor has proven its ability to withstand the loads associated with short-duration hypersonic testing in a simple spherical configuration [17]. On models with long wingspans or other thin geometry where high loading could present issues, the PF resin could be used as a skin over a metal substructure.

PF resin can be used as a standalone sensor, or it can be coated with luminescent material to produce a dual wavelength sensor. To develop the sensor, two types of PF resin, one with a paper base material and the other based on cloth fibers, were tested to determine which composition makes the most effective sensor. These laminate materials were selected for their low thermal conductivities. The characteristics of PF resin are reviewed further by Kim et al. [18]; the properties of other phenolic resin compounds have also been the subject investigation [19,20,21].

## 2. Materials and Methods

### 2.1. Sample Properties

**Figure 1:**
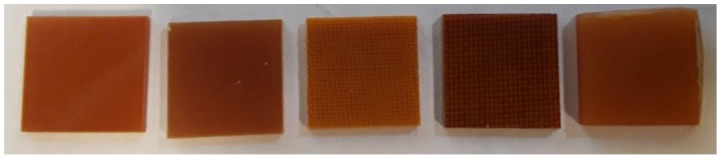
Phenolic resin samples. From left to right: P1, P2, C1, C2, and C3.

Two primary ways of manufacturing the resin involve either a cloth base or a paper/wood fiber base to provide strength to the composite resin [22,23]. The resin is formed into a phenolic sheet, the form of PF resin used in this experiment, when the uncured synthetic resin is impregnated into a laminate (cloth or paper). The resin polymerizes under the addition of heat and pressure, resulting in a hardened plastic sheet as a result [24]. The polymerized resin is the element of the material that provides luminescent output. Samples of both types are examined in this study to determine if either type exhibits optimal performance characteristics. Paper-based samples are denoted with a P, and cloth-based samples with a C. Samples for this experiment were obtained from Futamura (P1, C3), Nikkokasei (C2), and Risho (P2, C1); the differing samples with the same binder correspond to different manufacturers. The samples were 10 mm × 10 mm × 1 mm, shown in Figure 1.

### 2.2. Temperature Characterization

To characterize the samples, the optimal emission and excitation wavelengths were determined. This was done using a Fluoromax-4 spectrometer from Horiba (Kyoto, Japan) with a variable wavelength excitation source by sweeping through a range of excitation wavelengths to find an optimal point for each sample, then sweeping through a range of emission wavelengths to characterize the emission spectra of the samples.

**Figure 2:**
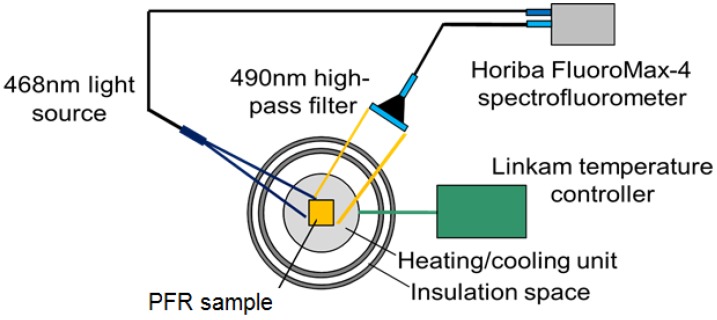
Static response testing setup.

A THMS600-PS temperature-controlled chamber from Linkam Scientific Instruments (Tadworth, United Kingdom) was used to study the static temperature response of the samples. Under 468 nm excitation, a spectrometer with a 490 nm high-pass filter was used to capture the spectral luminescent output of the PF resin samples at different temperatures, increasing to 500 K incrementally. Based on theory and previous TSP studies using diverse types of luminophores, the emission intensity was expected to decrease as temperature increases [14,25]. This characterization setup has been used by Sakaue et al. to study TSP on anodized aluminum and is shown in Figure 2. The static calibration of the PF resin samples was performed in 20 K increments, increasing from 100 K to 500 K. The temperature is measured at the interface of the sample and the heating/cooling element, so the sample was given five minutes at each temperature step to allow for the entire sample to achieve uniform temperature before the measurement was taken. The measurement chamber is initially purged of air and then pressurized to the measurement condition using dried air to prevent condensation at lower temperatures.

## 3. Results

### 3.1. Emission and Excitation

**Figure 3:**
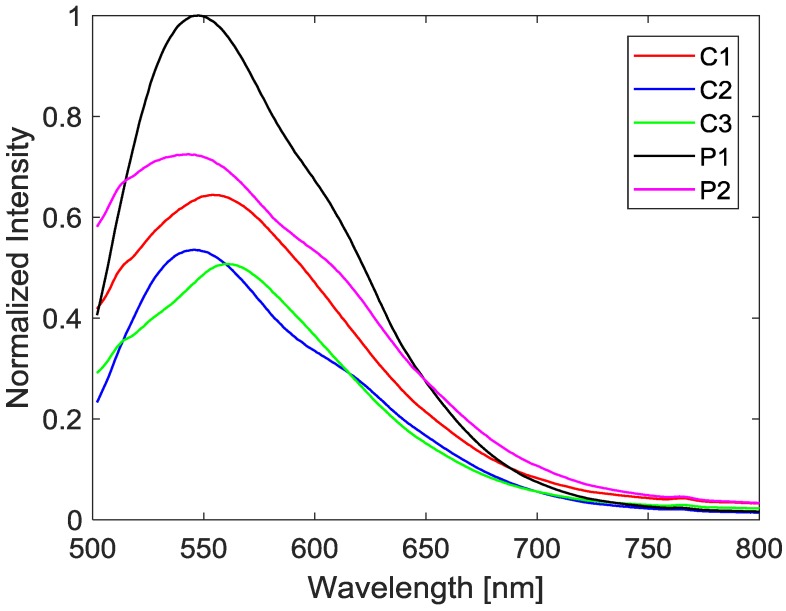
Spectral output of different PF resin samples at 300 K, normalized by the maximum intensity of the brightest sample, P1.

All samples produced the highest emission intensity when excited at a wavelength of 468 nm. At this excitation wavelength, all samples produced peak emissions in the range of 540–560 nm, which allows for the use of a 490 nm high-pass filter to filter out the excitation light and capture only the output signal. In practice, a bandpass filter can alternatively be used instead of a high-pass filter to limit the introduction of noise into the measurement. The spectral outputs of the samples at ambient temperature are shown in Figure 3, normalized by the maximum intensity of sample P1, which was the brightest sample at 300 K.

### 3.2. Luminescent Intensity

**Figure 4:**
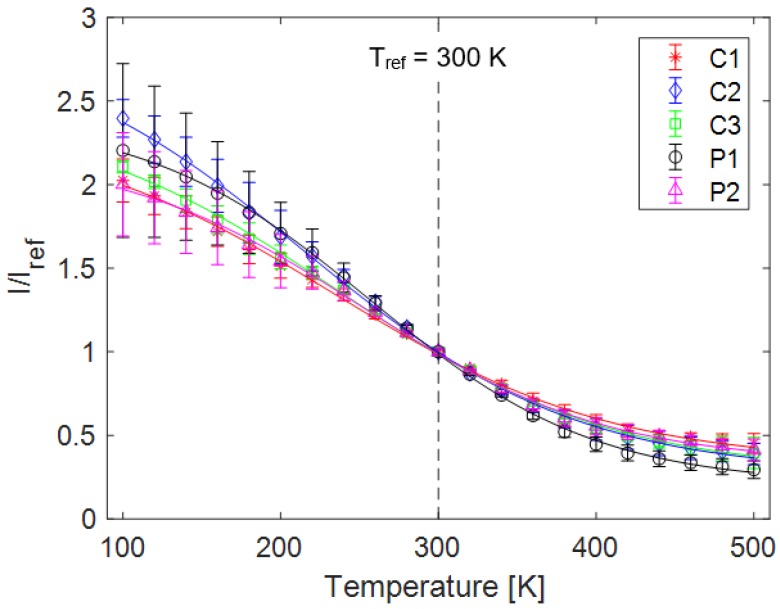
Emission intensity of each sample normalized by that sample’s intensity at 300 K.

As expected, the emission intensity of the samples decreased with increasing temperature, but still maintained sensitivity even at the highest temperature, as seen in Figure 4. The intensity curves of the samples display good self-similarity with increasing temperature when normalized by the maximum intensity at each temperature. The exception to this trend was a slight red shift in samples P1 and C2; a red shift is an increase in peak emission wavelength, shifting it closer to pure red light. The maximum shift of the peak wavelength was 5 nm in these two samples, which for a sufficiently large integration range is easy to account for.

The spectral data was used to determine a single value for emission intensity at each temperature, based on an integration region of 525–575 nm. When used for experimental measurements, a bandpass filter can be used to capture only the emission within this band. These values were then normalized by the intensity of each respective sample at room temperature to produce intensity curves over the measured temperature range. Monotonic decrease in intensity with increasing temperature is noted in all samples. From this, sigmoidal curves as described by Equation (1) were fit to the data, shown in Figure 4 with error bars representing the standard deviation of three data sets. Larger error farther from the reference condition is due to reduced ability of the controller to maintain a constant temperature, with uncertainty rising from ±0.1 K at 300 K to almost ±2 K at cryogenic conditions.

(1) $$\frac{I}{I_{ref}}=a_{0}+\frac{a_{1}}{1+\exp\left(-\frac{T-a_{2}}{a_{3}}\right)}$$

where *T* is temperature, *I* is luminescent intensity, and *a* indicates a constant. The subscript *ref* indicates the conditions at the reference temperature and pressure of 300 K and 100 kPa, respectively. The data can also be normalized by the intensity of the brightest sample (P1) at room temperature to put the changes in intensity into perspective; this analysis showed that sample P1 had the highest emission intensity while also being the most sensitive to temperature. This is useful because both emission intensity and temperature sensitivity are important characteristics of a temperature sensor. A brighter sensor requires shorter exposure times and is therefore more useful as a dynamic sensor. It was seen that a thicker sample produced a brighter emission, which indicates that the luminescence of PF resin is not purely a surface phenomenon.

### 3.3. Temperature Sensitivity

The emission intensity spectra were then used to determine the temperature sensitivities of the samples. Taking the derivative of Equation (1) yields a value for the temperature sensitivity of each sample throughout the temperature range, and the equation for determining this value is shown in Equation (2).

(2) $$\delta T={\frac{d\left(I/I_{ref}\right)}{dT}|}_{T=T_{ref}}=\frac{a_{1}}{a_{3}}{\left(\exp\left(\frac{T_{ref}-a_{2}}{2a_{3}}\right)+\exp\left(-\frac{T_{ref}-a_{2}}{2a_{3}}\right)\right)}^{-2}\;\left(\%/K\right)$$

This value represents the amount that the luminescent output changes for each degree of temperature change in Kelvin. A higher absolute value indicates a more sensitive sensor, and the sign on the temperature sensitivity simply indicates whether the intensity of the emitted light increases or decreases with temperature. Temperature sensitivities and working ranges are provided for three typical TSPs in Table 1.

**Figure 5:**
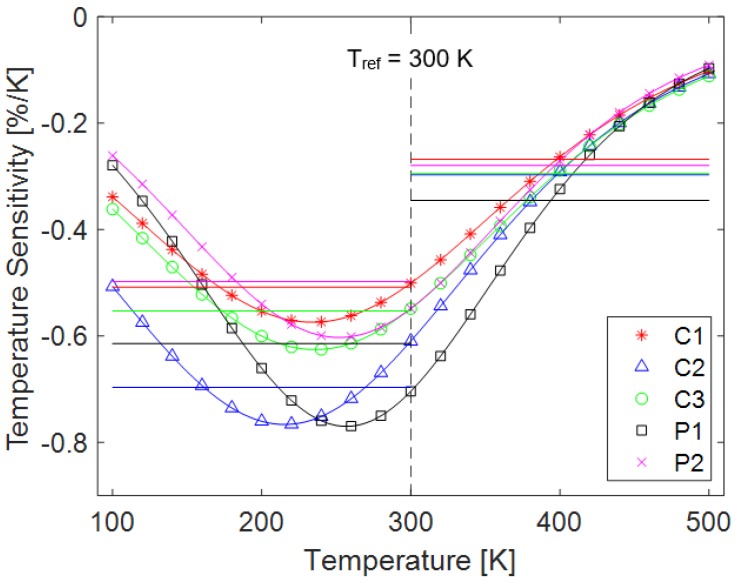
Temperature sensitivity of each sample based on a sigmoidal fit and a as a constant value based on a linear fit in regions above and below the reference condition.

The maximum temperature sensitivity of approximately −0.8%/K is achieved by samples C2 and P1 at 210 K and 260 K, respectively, based on the derivative of the sigmoidal fit, shown in Figure 5. All samples were most sensitive below ambient temperature, which is consistent with prior TSP studies [14,26]. It is often useful for engineering applications to use a constant sensitivity value for a range of temperatures, and by fitting linear regions to the intensity curve, constant values can be found for different temperature ranges. Two temperature ranges were selected: a low temperature region from 100–300 K, and a high temperature region from 300–500 K. The constant temperature sensitivity values are shown in Table 2 along with the goodness of the linear fits, indicated by the coefficient of determination (R^2^ value).

Based on these linear fits, different constant temperature sensitivities can be used in different temperature regimes. Above 300 K, all samples tested showed linear temperature sensitivities of approximately −0.3%/K. In the low temperature region, the sensitivity varied based on the sample between −0.5 and −0.7%/K, with sample C2 having the highest sensitivity.

### 3.4. Pressure Sensitivity

**Figure 6:**
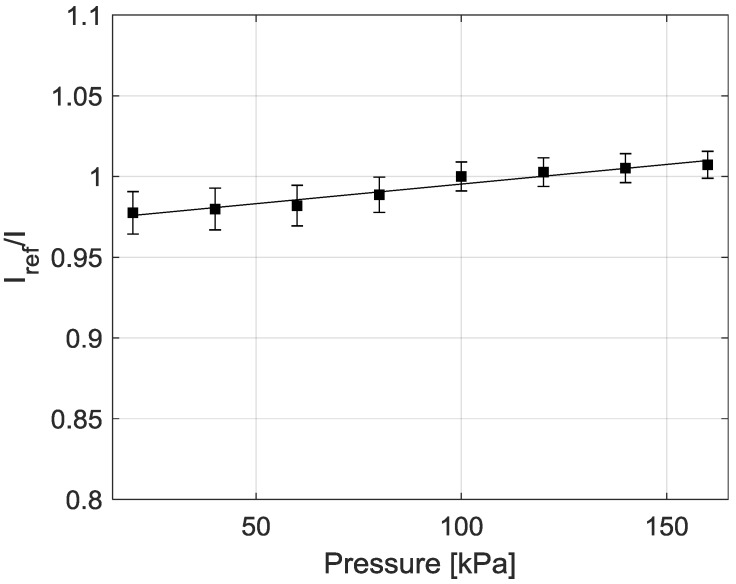
Stern-Volmer plot showing the pressure sensitivity of PF resin (sample P1).

As is typical of various TSP formulations, PF resin demonstrates a low sensitivity to changes in pressure. Figure 6 shows a Stern-Volmer plot of sample P1; these plots are used to show the pressure sensitivity of a sensor. Pressure and luminescent intensity are related by the Stern-Volmer equation, given by Equation (3).

(3) $$\frac{I_{ref}}{I}=A\left(T\right)+B\left(T\right)\frac{P}{P_{ref}}$$

where *P* is pressure, *T* is temperature, *I* is luminescent intensity, and a the subscript *ref* represents a reference condition, in this case 100 kPa and 300 K. *A*(*T*) and *B*(*T*) represent the Stern-Volmer coefficients, which are a function of temperature; a larger value of *B*(*T*) indicates greater pressure sensitivity [1]. Sample P1 was tested to determine the pressure sensitivity of PF resin. The Stern-Volmer coefficient *B*(300 K) was found to be 0.024. For comparison, EuTTA, another temperature-sensitive luminophore, applied in three different binder materials (PMMA, PEMA, and PBMA) exhibited *B*(*T*) coefficients of 0.056, 0.070, and 0.050, respectively [12].

## 4. Discussion

These calibration and static characterization results show that the PF resin samples perform comparably to similar traditional TSPs, with a maximum temperature sensitivity of just under −0.8%/K. While this is on the lower end of other temperature sensors, this type of sensor has many advantages over a traditional TSP. Taking these other advantages into consideration shows PF resin to be a very effective measurement tool. These advantages include the natural luminescent properties of PF resin, resistance to thermal expansion, durability, and the fact that no additional coating is necessary on a model, allowing for direct temperature measurements of the model surface. Additionally, this study found that there is no strong correlation between performance and laminate material, indicating that laminates with similar thermal conductivities do not impact the performance of the sensor. The differences in emission intensity are generally within the bounds of measurement error, which further supports the finding that the laminate material, in this case paper or cloth, does not significantly alter the temperature sensitivity or signal level of the sensor. Furthermore, the sensor has been effectively used to measure temperature in an aerodynamic heating test performed by Nakamoto et al. [17].

## 5. Conclusions

Advanced optical-chemical temperature sensors are poised to change the way that heat transfer is experimentally measured, allowing areas of high thermal stress and surface heat flux to be identified on a model. This study shows the characterization of a temperature sensing material, and found the highest sensitivity to be −0.8%/K at 210 K and 260 K. Using a linear fit to create regions of constant sensitivity, the highest sensitivities are −0.7%/K in the region from 100–300 K, and −0.3%/K in the region from 300–500 K. These values are important for engineering applications as they allow a constant value of temperature sensitivity to be used, instead of having to change the value of sensitivity with changing temperature (the quantity being measured). All samples are more sensitive in the region below 300 K. One of the paper-based samples shows the highest luminescent output at all temperatures, making it a viable candidate for further study, especially because it maintains sensitivity over a wide temperature range. The phenol-formaldehyde resin sensor developed provides a convenient way to produce a physical model and obtain global surface temperature data without adding additional paints or instrumentation to the surface. The sensor also provides sensitivity over a wide temperature spectrum, making it suitable for use in different temperature regimes.

## Figures and Tables

**Table 1:** Typical temperature-sensitive paint (TSP) sensitivities [1].

TSP	Temperature Range [°C]	Temperature Sensitivity [%/°C]
EuTTA in dope	−20 to 80	−3.9
Ru(bpy) in shellac	0 to 90	−0.93
Ru(trpy) in GP-197	−170 to −50	−1.34

**Table 2:** Temperature sensitivities, *δT*, and corresponding R^2^ values.

	Low (100–300 K)		High (300–500 K)		Sigmoidal	
	***δT***	**R^2^**	***δT***	**R^2^**	**Maximum *δT***	**R^2^**
**C1**	−0.5	0.99	−0.3	0.90	−0.6	0.99
**C2**	−0.7	0.99	−0.3	0.92	−0.8	0.99
**C3**	−0.6	0.99	−0.3	0.92	−0.6	0.99
**P1**	−0.6	0.99	−0.3	0.90	−0.8	0.99
**P2**	−0.5	0.99	−0.3	0.91	−0.6	0.99
